# Elevated Foxp3/CD8 Ratio in Lung Adenocarcinoma Metastatic Lymph Nodes Resected by Transcervical Extended Mediastinal Lymphadenectomy 

**DOI:** 10.1155/2017/5185034

**Published:** 2017-08-02

**Authors:** Joanna Domagala-Kulawik, Iwona Kwiecien, Juliusz Pankowski, Monika Pasieka-Lis, Dominika Wolosz, Marcin Zielinski

**Affiliations:** ^1^Department of Internal Medicine, Pulmonology and Allergology, Medical University of Warsaw, Warsaw, Poland; ^2^Laboratory of Flow Cytometry and Hematology, Department of Internal Medicine and Hematology, Military Medical Institute, Warsaw, Poland; ^3^Department of Pathology, Pulmonary Hospital, Zakopane, Poland; ^4^Department of Pathology, Medical University of Warsaw, Warsaw, Poland; ^5^Department of Thoracic Surgery, Pulmonary Hospital, Zakopane, Poland

## Abstract

A balance between tumor invasion and immune defence system is widely investigated.* Objective*. The aim of this study was to evaluate lymphocyte phenotype in lymph nodes (LNs) of patients with lung cancer in relation to the presence of metastases.* Methods*. We investigated 364 LNs resected by transcervical extended mediastinal lymphadenectomy (TEMLA) of 49 patients with squamous cell carcinoma (SCC) or adenocarcinoma (AD) with (A) and without metastases (B). Expression of CD4, CD8, CD25, CTLA-4, and Foxp3 was assessed by immunohistochemical staining.* Results*. We observed a strong nuclear staining for Foxp3 in lymphocytes and cancer cells and strong membranous/cytoplasmatic reaction for CD4 and CD8, but low for CD25 and CTLA-4. There were significantly higher proportions of CD8+ cells in AD (B) versus AD (A) LNs (80% versus 52.5%, *p* < 0.05). The Foxp3/CD8 ratio was higher in AD (A) versus AD (B) LNs (0.4 versus 0.25, *p* < 0.05). No significant differences in the cell markers expression in SCC LNs were found.* Conclusion*. Significant differences in lymphocyte phenotype in AD may indicate an exceptional biology of this type of lung cancer. TEMLA resected LNs may serve as valuable samples for evaluation of immune status in lung cancer patients.

## 1. Introduction

Lung cancer remains a leading cause of cancer death. An incidence is as high as 1.800.000 new cases yearly worldwide; the number of deaths reaches 1.600.000 globally [1]. This epidemiology is not improving in spite of some efficacy of the antitobacco activities. Lung cancer is a disease of advanced age and those patients are the victims of epidemic of smoking in the last decades of XX century. On the other hand, the growing incidence of lung adenocarcinoma among never smoking women is observed [2]. The clinical course of lung cancer is changing; the prolonged patients survival is observed. The improving methods of accurate cancer diagnosis and new treatment options contribute to this change. Some lung adenocarcinoma patients benefit from targeted therapy. More and more patients in the advanced stages of the disease may benefit from immunomodulatory treatment [3, 4]. To date the antiprogrammed death (PD-1) blockers are approved in patients with a positive immunohistochemistry staining for PD-L1 of squamous and nonsquamous histology [5]. New immunomodulatory agents were found to be effective in ongoing trials [3]. The main goal of this kind of therapy is to reactivate host anticancer immune response. Cancer immunotherapy seems to be a method of precision medicine and the biomarkers which may serve as predictive factors are widely investigated. The status of patients' immune system before treatment is highly individual and influenced by intrinsic and external agents, like tobacco smoke, comorbidities (e.g., chronic lung diseases like COPD), and drugs [6]. This status of immune system before treatment is very important and may be assessed by peripheral blood or cellular infiltration in tumor microenvironment (TME) analysis. The resection rate in lung cancer is as low as 20–30%, so TME is not available for analysis in most of the cases. An immune reaction to cancer is created in lymph nodes [7]. Antigen presenting cells in lymph nodes are able to activate cytotoxic lymphocytes after recognition of cancer antigens. Thus, lymphocyte phenotype in regional lymph nodes could be useful to characterize the background of immune response. The population of lymphocytes may be simply divided into two types: CD4+, CD8+, and natural killer (NK) cells exhibiting the function of cytotoxic cells and CD4+/CD25+/Foxp3+ cells, which form a regulatory population capable of inhibiting anticancer immune response [7]. CTLA-4 is a strong suppressive molecule expressed on T effector cells and on T regulatory cells, as well [8]. Therefore, we choose the following antibodies to characterize lymphocyte phenotype of LNs: anti-CD4, anti-CD8, anti-CD25, anti-Foxp3 and anti-CTLA-4 in our study. Recently the availability of whole lymph nodes for examination becomes limited; surgery techniques were replaced by less invasive method: endobronchial ultrasound guided fine needle aspiration (EBUS/TBNA) [9]. We have an access to the whole LNs resected by transcervical extended mediastinal lymphadenectomy (TEMLA) [10] and in this study we have compared the lymphocyte phenotype in TEMLA resected LNs group seven with and without lung cancer metastases.

## 2. Methods

The analysis was performed on LNs station seven resected by TEMLA from patients with lung cancer during the diagnostic and staging process. Appropriate patient consent was received before surgical procedure. The TEMLA method of LNs exploration was previously described in detail [11]. The conventional light microscopy and hematoxylin-eosin staining were used for lymph nodes examination. The group of LNs was divided into those in which metastatic cells were visualized (A) and those without metastases (B). The LNs group seven of patients with two main histological types of lung cancer, squamous cell carcinoma (SCC) and adenocarcinoma (AD), were selected. The clinical data and the number of LNs were presented in Table 1. This study was performed during diagnostic process and there was no data about the kind of treatment and treatment results.

For lymphocyte phenotyping by immunohistochemistry (IHC) the following antibodies were used:

- CD4 (anti-CD4 Rabbit Monoclonal Primary Antibody, Ventana Medical Systems)
- CD8 (anti-CD8 Rabbit Monoclonal Primary Antibody, Ventana Medical Systems)
- CD25 (anti-CD25 Rabbit Monoclonal Primary Antibody, Ventana Medical Systems)
- CD152/CTLA-4 (Polyclonal Rabbit Primary Antibody anti-CD152/CTLA-4, ThermoFisher Scientific)
- Foxp3 (Mouse Monoclonal Primary Antibody anti-Foxp3, BD Pharmingen™)

Antibodies anti-CD4, anti-CD8, and anti-CD25 are ready to use for automatic staining. Only the original antibodies anti-CD152 and anti-Foxp3 were diluted in 1 : 100 ratio using Antibody Diluent (Ventana Medical Systems). IHC staining was performed automatically on BenchMark Classic (Ventana Medical Systems) with NexES computer system according to the instruction of manufacturer. The appropriate control slides were used. For visualization the diaminobenzidine tetrahydrochloride (DAB) was used.

The evaluation of LNs phenotype was performed independently by two pathologists. Firstly, all samples were analyzed in light microscopy, under 200x magnification, and the general pattern of each lymph node was noted. Next, the areas of high number of CD4+ cells were marked on the slides with anti-CD4 staining. In the following step, the same parts were selected in each lymph node on the slides with reaction to CD8, CD25, Foxp3, and CTLA-4, respectively. Finally, five different fields in these areas were chosen and 300 cells in each field were counted under 400x magnification. The intensity of reaction was estimated subjectively as +, ++, and +++. The proportion of positively stained cells in each field was counted. A special attention was paid to lymphocytes adjacent to cancer cells in metastatic LNs. The results were presented as a proportion of positively stained lymphocytes of all lymphocytes.

**Figure 1:**
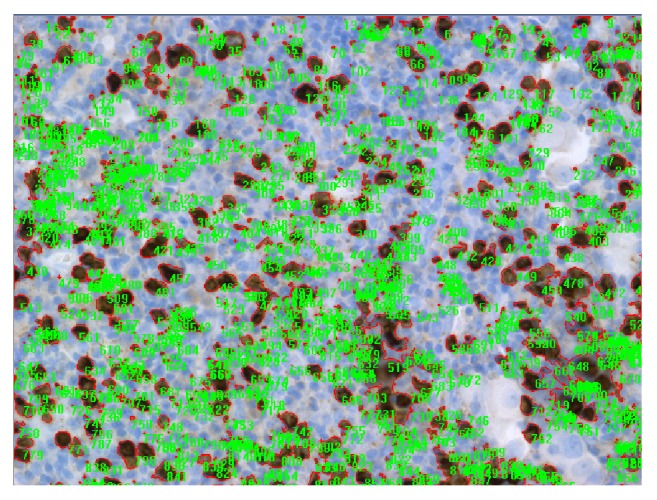
Example of automated analysis of CD8+ cells in lymph node resected by TEMLA.

Automatic method was employed in cell counts validation. After immunostaining, slides were analyzed in light microscopy on Nikon Eclipse 80i (Nikon, Japan) under 200x magnification in five fields of view and recorded by QICAM Fast 1394 camera. Next, data was analyzed by Image Pro Plus program (Media Cybernetics, USA). In this method the cells were counted and recorded as an arbitrary unit = 255 − immunoreactivity × area. An example of this analysis was presented in Figure 1.

The ratio of the percentage of Foxp3 positive cells to the percentage of CD8 positive cells was calculated for each LN.

### 2.1. Statistical Analysis

We used Statistica 10.0 software (StatSoft, United States of America) for statistical analysis of collected data. Kruskal-Wallis test was performed to analyze the differences between the groups of patients. For correlation analysis we used the Spearman rank correlation coefficient. We considered *p* value < 0.05 as statistically significant.

## 3. Results

**Figure 2:**
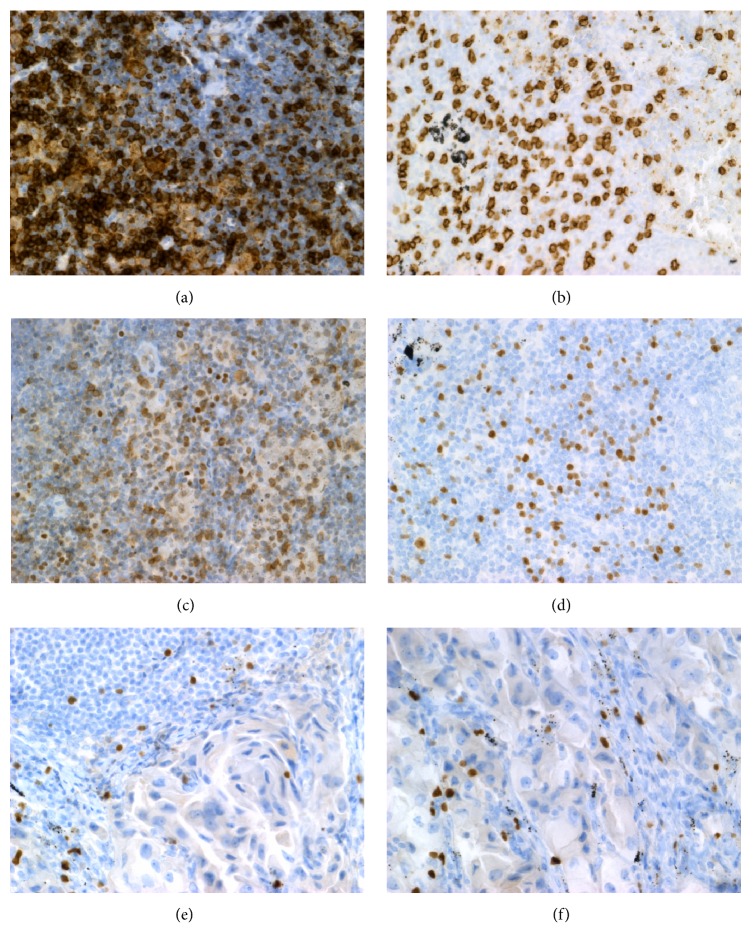
Images of immunostaining of lymphocytes in lymph nodes resected by transcervical extended mediastinal lymphadenectomy (TEMLA) of patient with lung cancer (×400). (a) CD4+ cells, (b) CD8+ cells, (c) CD25+ cells, (d) Foxp3 positive lymphocytes, (e) Foxp3 positive lymphocytes adjacent to cancer cells, and (f) positive reaction of Foxp3 in cancer cells.

**Figure 3:**
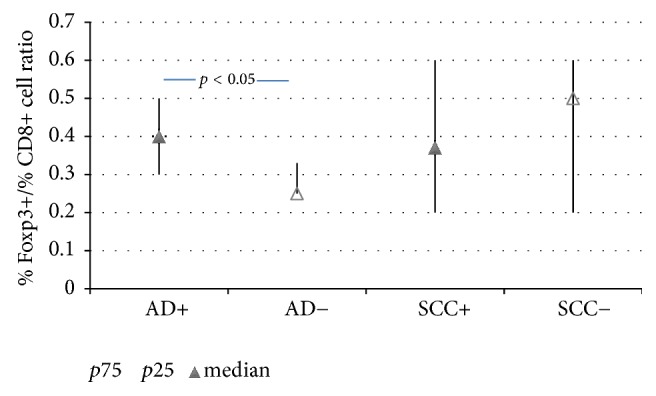
A ratio of the proportion of Foxp3+ cells to CD8+ cells in the lymph nodes resected by TEMLA of patients with squamous cell type (SCC) lung cancer and lung adenocarcinoma (AD) with (+) and without (−) detected metastases. Data expressed as median value (*p*25–*p*75).

We observed the positive reaction with antibodies anti-CD4, anti-CD8, anti-CD25, and anti-Foxp3. In general the reaction for these T cell markers was negative in germinal centers of LNs. Only sparse cells were positive to CD152 (CTLA-4) in all LNs so this marker was excluded from further analysis. We observed the following types of reaction: cytoplasmatic/membranous for CD4, CD8, and CD25 and nuclear for Foxp3 (Figure 2). The highest intensity and percentage revealed CD4+ cells: the intensity was “+++” and a proportion was 90–100% and did not differ between investigated groups of LNs. For CD8+ cells the mean intensity of the staining was also high, “+++”. There was significant correlation between the intensity of CD4+ staining with cell proportion and between CD8+ staining with cell proportion (*r* = 0.40, *p* < 0.05 and *r* = 0.36, *p* < 0.05, respectively). The proportion of CD8+ cells was significantly higher in LNs from adenocarcinoma patients without metastases when compared to LNs from adenocarcinoma patients with metastases (median value (*p*25–*p*75): B = 80% (60–80) versus A = 52.5% (50–60), *p* < 0.05); see Table 2. The intensity of CD25 reaction was “+” to “++” and the proportion of CD25+ cells was comparable between the groups. The intensity of nuclear Foxp3 reaction was “+” to “+++” and the proportion of Foxp3 positive cells was comparable between the groups. The Foxp3+/CD8+ ratio was significantly higher in LNs with metastases of adenocarcinoma when compared to the negative LNs (median value, (*p*25–*p*75): A = 0.40 (0.30–0.50) versus B = 0.25 (0.25–0.33), *p* < 0.05); see Figure 3. We noticed a positive reaction for Foxp3 not only in lymphocytes but also in some nuclei of cancer cells. The augmentation of Foxp3 positive lymphocytes in the fields adjacent to cancer cells was observed (Figure 2).

## 4. Discussion

The aim of our study was to evaluate lymphocyte profile in lymph nodes affected by cancer in the course of lung adenocarcinoma and squamous cell carcinoma versus lymph nodes free of metastases. We have investigated for the first time a large number of lymph nodes resected by TEMLA. We found a significantly lower proportion of CD8+ cells and a significantly higher Foxp3+/CD8+ ratio in LNs affected by adenocarcinoma versus LNs free of metastases.

There is growing body of evidence that the evaluation of host immune status in patient with solid tumor is very important and may serve as a prognostic factor in addition to routine TNM classification. Lymphocyte phenotype is accepted as an important feature of the character of anticancer immune response. It was pointed out by Galon et al. who described a method of immunoscoring in colon carcinoma [12], recently it was described by Blank et al. as an element of cancer “immunogram” [13]. Donnem et al. in the large study on about 800 non-small-cell lung cancer (NSCLC) patients concluded that the density of CD8+ cells in tumour infiltrating lymphocytes (TIL) is a strong independent factor for such clinical responses, like disease-free survival and overall survival [14]. Senovilla et al. and Ogino et al. emphasized a role of the analysis of T cell repertoire as possible immune prognostic/predictive factor in cancer [15, 16]. Two opposite populations could be identified among TIL: anticancer lymphocytes (cytotoxic T lymphocytes, CTLs): CD4+, CD8+, NK cells, and regulatory T cells (Tregs: CD4+/CD25+/Foxp3+ cells) as well as lymphocytes with CTLA-4 expression. Thus the CTLs/Tregs balance needs special attention [4, 17] and our study fit into this trend.

The full analysis of TIL is possible only in a small number of lung cancer cases which are qualified to resection. Even in these cases, the inflammatory reaction may be difficult to quantitative analysis because of the different intensity of immune infiltrations and the difficulties in the whole tissue preservation during processing. The immune response may be evaluated by peripheral blood examination, but it reflects systemic changes which are different from local one. For deeper and specific characterization of local immune response, the analysis of bronchoalveolar lavage fluid (BALf) may be used [18]. However, such usefulness of BALf examination was not validated to date.

The anticancer reaction is created in regional LNs and their samples may be useful in cancer immunological studies [15, 19, 20]. There are some different methods of LNs examination in the process of the lung cancer staging. Recently, endobronchial ultrasound with transbronchial needle aspiration (EBUS/TBNA) was acknowledged as superior to surgical methods [9]. The cytological material obtained by TBNA, even when analyzed by flow cytometry or cytoblock technique gives only selective, not complex lymphocyte phenotypical pattern. In this study we have had an access to unique samples of LNs which were resected by TEMLA. These LNs are fully accessible to evaluation. Recently, in the era of the development of lung cancer immunotherapy the LNs harvested by TEMLA could be valuable objects for the investigation of the biomarkers for this therapy. To the best of our knowledge our study is the first in which these LNs were used for lymphocyte phenotyping. We have chosen station seven which is the most important in evaluation of N2 involvement. TEMLA was adopted also for resection of lung cancer. The analysis of basic T lymphocyte markers, CD4 and CD8, was shown to be useful in the prognosis for survival in lung cancer after surgery [12, 14, 21–23]. Thus, the new prognostic factor could be achieved by adding the simplified IHC lymphocyte phenotyping to the routine examination of LNs resected by TEMLA during surgical treatment of lung cancer.

The quantitative analysis of cell phenotype is a challenge for pathologists. In many studies the manual methods were used but, with the risk of error of subjectivism. Some authors present their results as a grading of positive stained cells with the following cut-off values: 0-5-25-50% [14, 22]. Donnem et al. recommended a cut-off at 25% and 50% positive cells as easy to reproduce marker [14]. In all cohorts in this multicentre study, the high agreement of distinction between low, intermediate, and high density of cells was confirmed. Many new automatic methods were described and a concordance with manual methods was shown [24–27]. Our study also confirmed this concordance. However, as it was shown in the evaluation of programmed death ligand (PD-L1) before anti-PD-1 therapy [28] a uniform analytical system does not exist and its construction is needed. In our study we present our results as an absolute proportion of positive cells, which was calculated manually among all lymphocytes in the fields with their high density. We achieved a good concordance between two pathologists and with an automatic method. Our results also show that the immune response is highly individual. There were LNs with low or high proportion, in particular, of CD8+ cells. Thus the presentation of the results as a ratio of the proportion of cells seems to be most objective. Consistent with Schneider et al., we found a high proportion of positive lymphocytes in the fields adjacent to tumor nests in LNs [29].

As mentioned above, we used the classic T cell markers to show the CTLs/Tregs balance. The high proportion of CD4+ cells in LNs, and no differences between the groups could be expected. The proportion of CD8+ cells was high with high intensity of reaction and was higher in LNs free of metastases when compared with affected LNs. It was confirmed by the results of other studies in which the high proportion of CD8+ cells in TIL was shown to be favorable prognostic factor in solid tumors [14, 30, 31].

CD25 is a marker of T cell activity and serve as one of the markers of Tregs. CD25 is recently investigated as a target for antiregulatory T cell therapy [32]. In our study the reaction with anti-CD25 antibody was easy to identify (Figure 2). However, no differences in the proportion of CD25+ cells in LNs of different groups of patients were found. A role of CD25 as a biomarker needs further investigation.

CTLA-4 was chosen by us as a marker of the suppression of anticancer response and as a possible biomarker for checkpoints blockers [3, 8]. We did not observe any relevant positive reaction, possibly because of the prevalence of intracellular expression of this molecule and technical problems with detection [8]. Flow cytometry seems to be a better method than IHC for CTLA-4 detection [33].

Foxp3 is a transcription factor with very strong suppressive properties [17]. An elevated expression of Foxp3 was shown to be unfavorable prognostic factor in solid tumors [21, 22, 34, 35]. The Foxp3/CD8 ratio reflects (in brief) the balance between suppression and activation of anticancer immune response. The median proportion of Foxp3+ lymphocytes in our study was 20–30%. We detected expression of Foxp3 also in some nuclei of cancer cells. The weakness of our study is the use of only a simple staining method without possibility of precise location of Foxp3. Using in the future a double staining (for example in confocal microscopy) may lead to improving the recognition of the cells with Foxp3 expression and recognition of Treg cells. Tao et al. defined Foxp3 positive tumors when at least 20% of the cells were positive to this marker with some implication to the prognosis [21]. Liu et al. analyzed expression of Foxp3 in tumor and TIL and found that only the expression of Fopx3 in TIL plays a role of prognostic factor [36]. Their study confirmed the importance of high Foxp3/CD8 ratio as a predictor of poor response to chemotherapy. The TIL Foxp3/CD8 ratio in their study was comparable to our results. Petersen et al. found that patients with high intratumoral Foxp3/CD3 ratio had high risk of recurrences of stage I lung cancer after surgery [22]. Thus, the low proportion of CD8+ cells may indicate an inadequate ability to mobilization of anticancer cytotoxicity in LNs. The high Foxp3/CD8+ ratio reflects a predominance of suppression over activation of immune system. It may be presumed that such “suppressive” pattern of metastatic LNs is the result of initially impaired anticancer immunity in aggressive tumour.

We observed significant differences of Foxp3/CD8 ratio between metastatic versus free LNs only in adenocarcinoma. Schneider et al. analyzed lymphocyte phenotype in regional LNs by flow cytometry and their study also revealed important accumulation of Tregs in metastatic LNs in adenocarcinoma but not in SCC [29]. Anticancer immune response is to date investigated in NSCLC rather without regard to histological types. However, there is evidence that adenocarcinoma significantly differs from SCC in many aspects and is a more complex disease than SCC [37]. There is a prevalence of adenocarcinoma in women and a smaller relationship with tobacco smoking in adenocarcinoma than in other types of lung cancer is observed in the epidemiological studies. There are well defined molecular alterations in patients with adenocarcinoma being a basis for targeted therapy. Finally, the recognition of adenocarcinoma subtypes which differ in morphology, in the clinical course, and in molecular characteristic confirms the unique character of this neoplasm. Our results contribute to consider the specificity of adenocarcinoma also in the aspect of the immunity.

## 5. Conclusion

The immunohistochemical analysis of lymphocyte phenotype in the whole LNs resected by TEMLA provides a valuable information of the host immune status of lung cancer patient. The lower proportion of CD8+ cells and higher Foxp3+/CD8+ ratio in metastatic when compared with nonmetastatic LNs in adenocarcinoma may indicate a particular biology of this type of lung cancer.

## Figures and Tables

**Table 1:** Characteristics of the study group. AD: adenocarcinoma, SCC: squamous cell carcinoma, “+”: lymph nodes with metastases, “−”: without metastases, and LNs: lymph nodes.

Histological type	Patients *number *(F/M)	Age *mean (range)* [years]	LNs *total number*	LNs *mean number/person *(range)	Metastases *number*	Metastases *mean number/person *(range)
Squamous cell carcinoma						
A (SCC+)	12 (1/11)	58.6 (44–76)	92	7.66 (3–14)	32	2.66 (1–7)
B (SCC−)	18 (4/14)	64.4 (52–81)	141	7.8 (4–12)	0	0
Adenocarcinoma						
A (AD+)	12 (5/7)	53.6 (45–76)	86	7.1 (4–10)	42	3.5 (1–7)
B (AD−)	7 (3/4)	57 (48–68)	45	5.3 (4–12)	0	0

**Table 2:** Proportion of CD8+, CD25+, and Foxp3+ cells in the lymph nodes resected by TEMLA of patients with squamous cell type (SCC) lung cancer and lung adenocarcinoma (AD) with (+) and without (−) detected metastases. Data expressed as median value, (*p*25–*p*75). ^*∗*^Significant difference between groups A and B in Mann-Whitney *U* test, *p* < 0.05.

	CD8+ [%]	CD25+ [%]	Foxp3+ [%]
A (AD +)	52.5^*∗*^ 50–60	12.5 10–22,5	20 11–30
B (AD −)	80^*∗*^ 60–80	12 10–30	20 15–25
A (SCC +)	65 50–80	25 15–30	23.5 11–30
B (SCC −)	60 50–65	10 10–20	30 18–40
Intensity	+++/++	++/+	+++/+
